# Retained duplicate genes in green alga *Chlamydomonas reinhardtii* tend to be stress responsive and experience frequent response gains

**DOI:** 10.1186/s12864-015-1335-5

**Published:** 2015-03-04

**Authors:** Guangxi Wu, David E Hufnagel, Alisandra K Denton, Shin-Han Shiu

**Affiliations:** Cell and Molecular Biology Program, Michigan State University, East Lansing, MI USA; Department of Plant Biology, Michigan State University, East Lansing, MI USA; Biochemie der Pflanzen, Heinrich-Heine-Universität Düsseldorf, Düsseldorf, Germany

## Abstract

**Background:**

Green algae belong to a group of photosynthetic organisms that occupy diverse habitats, are closely related to land plants, and have been studied as sources of food and biofuel. Although multiple green algal genomes are available, a global comparative study of algal gene families has not been carried out. To investigate how gene families and gene expression have evolved, particularly in the context of stress response that have been shown to correlate with gene family expansion in multiple eukaryotes, we characterized the expansion patterns of gene families in nine green algal species, and examined evolution of stress response among gene duplicates in *Chlamydomonas reinhardtii*.

**Results:**

Substantial variation in domain family sizes exists among green algal species. Lineage-specific expansion of families occurred throughout the green algal lineage but inferred gene losses occurred more often than gene gains, suggesting a continuous reduction of algal gene repertoire. Retained duplicates tend to be involved in stress response, similar to land plant species. However, stress responsive genes tend to be pseudogenized as well. When comparing ancestral and extant gene stress response state, we found that response gains occur in 13% of duplicate gene branches, much higher than 6% in *Arabidopsis thaliana*.

**Conclusion:**

The frequent gains of stress response among green algal duplicates potentially reflect a high rate of innovation, resulting in a species-specific gene repertoire that contributed to adaptive response to stress. This could be further explored towards deciphering the mechanism of stress response, and identifying suitable green algal species for oil production.

**Electronic supplementary material:**

The online version of this article (doi:10.1186/s12864-015-1335-5) contains supplementary material, which is available to authorized users.

## Background

Green algae are a group of photosynthetic organisms that are more closely related to land plants than to other major eukaryotic groups [1]. A number of micro-green-algal species are suitable for biofuel production, and the lipid content of these algae increases significantly under various stress conditions [2]. For example, in the green algal model *Chlamydomonas reinhardtii*, lipid droplets rich in triacylglycerol (TAG) form after nitrogen (N) deprivation [3,4]. Other stress conditions, such as salt stress and sulfur deprivation, also lead to increased TAG content in *C. reinhardtii* [5,6]. In addition, stress response and subsequent lipid accumulation exhibit tremendous diversity in green algae [2]. For example, in response to N deficiency several green algae showed substantial differences in the tradeoff between growth and lipid content, the lipid accumulation time-course, and the stress level required to stimulate lipid formation [7]. Thus, a better understanding of the mechanistic details of stress response in green algae will not only contribute to our knowledge about adaptation to stressful environments but also will have the potential to improve microalgal biofuel production.

To better understand how green algae respond to stress on a genomic level, we focused on the retention and loss of gene family members. This is because gene duplications lead to raw materials for evolution to act on [8,9] and they are a common feature of most eukaryotic species [10]. Genes involved in stress response tend to be retained in various eukaryotes [11,12]. Thus a thorough look at the retention and loss patterns among green algal gene families may provide a new evolutionary perspective on stress response in green algal lineage. In general, the majority of gene duplicates are rapidly lost following the duplication events, but a significant number of duplicates are retained [10,13,14], contributing to organismal and regulatory complexity [11,15]. Gene retention occurs in a lineage-specific manner in a wide range of organisms [11,12,16] and a significant functional bias exists. In fungi, stress-related genes tend to undergo many duplications and losses [17]. Similarly, in *Arabidopsis thaliana*, stress responsive genes tend be retained [12] but also tend to be pseudogenized [18]. In a study of duplicates in yeasts, nematode, fruit fly, and *A. thaliana*, genes involved in response to environmental stress are prone to be retained in a lineage-specific manner [11]. There is not yet a global study summarizing the gene gain and loss patterns of known gene families in green algae. In addition, it is not known if duplicate retention is correlated with their stress responsiveness in green algae.

After duplication, gene duplicates may acquire a novel function that contributes to adaptation [8]. Such neo-functionalization can be an important source of inter-specific differences in stress response that could lay the foundation for diversity in stress-induced oil production in algae. An earlier study in *A. thaliana* showed that, although the predominant fate of gene duplicate was retention or loss of stress response, in around 6% of the cases there was evidence of stress response gain [19]. Examining evolution in gene expression among duplicates could thus further elucidate how gene duplication and subsequent functional innovation shaped the gene repertoire involved in stress response, and likely contributed to the diversity in stress response in green algae. There is no global study on functional evolution of duplicate genes in green algae.

In this study, we integrated genomic and transcriptomic data to find out how gene families and gene expression have evolved in the green algal lineage in the context of stress. We first examined the variation of domain family composition in nine green algal species compared to land plants. Then we investigated how gene gain and loss events occurred in the green algal lineage and examined the functional bias in retained duplicates in *C. reinhardtii* using phylogenetic approaches. We also examined the pseudogenization of gene duplicates and their functional bias. Finally, we characterized the evolution of gene expression after duplication events to find out how gene function evolved in the context of stress response. Our study reveals the evolutionary trajectory of stress responsive gene families in the green algal lineage, and because lipid production is sharply induced by stress [2], our results could help pinpointing candidate genes for further study on lipid metabolism.

## Results and discussion

### Variation in domain family sizes among land plants and green algae

**Figure 1:**
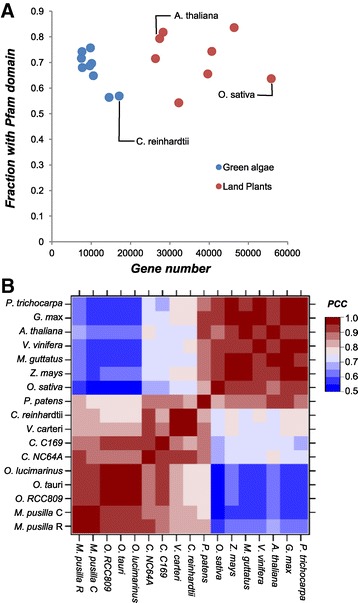
**Domain families in green algae and land plants. (A)**. The coverage of Pfam domain annotation in green algal (blue) and land plant (red) genomes. X axis indicates the numbers of genes in each species, and Y axis indicates the proportion of genes with ≥1 domains. **(B)**. Correlation of domain family size profiles among green algae and land plants. The color scale indicates the range of Pearson’s Correlation Coefficient (PCC) of domain family sizes between two species. Refer to methods for abbreviations.

Our goal here was to define the overall gain and loss of duplicates in gene families over the course of green algal evolution by using protein domains to define gene families. Gene families can be defined in two ways. In an earlier study, a protein sequence similarity based approach was used to identify protein families using full length protein sequences in green algae [20]. Here, we used another approach by defining a family as proteins having the same domain, using the Pfam database [21]; protein domains are well-defined regions of a protein that can perform a specific function and form a structural unit [22]. This approach was adopted because the sequence similarity approach could group non-homologous genes/regions into the same family (A is related to B, B to C, but not A and C). On the other hand, our approach only includes proteins with known domains. On average 65.7% green algal genes have one or more recognized domains, ranging from 56% to 76% among different species, while land plants have slightly higher percentage (Figure 1A, Additional file 1: Table S1). This could reflect the fact that overall green algae are less well studied than land plants and might have unknown algal-specific domains. It could also indicate a discrepancy in structural (gene) annotation quality between different green algal species. Given that genes with known domains are regarded as gene models with higher confidence [23], using protein domains to establish family alleviates the structural annotation quality issue.

Among 5,441 domain families found in nine green algae and eight land plants (see Methods), 3,897 (71.6%) are shared between green algae and land plants, 759 (13.9%) are green alga specific, and 785 (14.4%) are land plant specific. As expected, more closely related species tend to have more highly correlated domain family sizes and there are clearly two distinct clusters, one for green algae and the other for land plants (Figure 1B). Nonetheless, there is large variation in family sizes among green algal species. This is likely the results of lineage-specific gene gain and loss events. Thus we next asked how lineage-specific gene gains and losses have contributed to the extant domain family sizes in the green algal lineage.

### Gene gains and losses throughout green algal lineage

**Figure 2:**
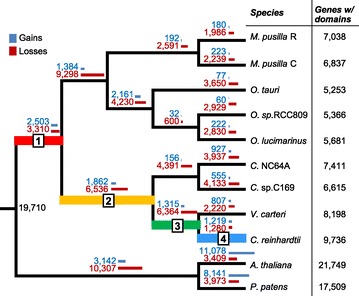
**Gene gains and losses across land plants and green algae.** Blue and red bars indicate number of the gene gain and loss events on each branch, respectively. Species names and numbers of annotated genes with domain in each species are shown on the right. Branch 1, 2, 3, 4 indicate four different time periods in the *C. reinhardtii* lineage evolution. Refer to Methods for full species names.

The variation in domain family sizes among green algae suggests extensive lineage-specific evolution of gene families. To find out how gene gain and loss events over time have shaped the domain family size differences among green algal species, we conducted a phylogenetic analysis on domain families present in green algae. To address the concern for gene annotation quality in green algae, we examined the current annotation and found that only a small number of recognizable protein domains are represented in intergenic regions (11% of domains found in annotated genes in *V. carteri*, 2-6% in other species, see Methods). Therefore, we used domain sequences in annotated genes for further analysis. Domain sequences from two land plants, *A. thaliana* and *Physcomitrella patens*, were included as outgroups. Among 4,656 domain families containing green algal genes, 4,207 with at least four sequences in green algae and the two land plants were further analyzed and a phylogenetic tree was built for each domain family. After reconciliation of the domain trees with a species tree [24], orthologous groups (OGs) among the green algal and the land plant species were established for inferring gene gain and loss events (Additional file 2: Figure S1B). We have also generated another species tree based on 18 s rRNA sequences (Additional file 3: Figure S5) and the two trees are largely similar, except in the *Ostreococcus* lineage. This ambiguity is likely due to the short branch length in this lineage that is difficult to resolve. This tree-based approach is shown to be consistent with similarity-based approach to identify OGs in land plants [12]. Overall, gene gain and loss events were frequent in every branch of the phylogenetic tree (Figure 2). Interestingly, in the green algal lineage, gene loss occurred more frequently than gene gain on every branch, suggesting extensive net gene loss since the green algae-land plant common ancestor.

Another reason for an excess of loss events could be because there were errors in phylogenetic inferences. To ascertain that our phylogenetic inference of orthologous groups was robust, we conducted two tests. First, we randomly picked 1,500 gene families for bootstrap studies. Among 50,690 branches, 46.5% have a bootstrap value of ≥ 80% (Additional file 4: Figure S3A). We found that 89.4% of the Volvocales branches (*C. reinhardtii* and *V. carteri*) have a bootstrap value of ≥ 80% but only 47.1% in the *Ostreococcus* branches (*O. sp*.RCC809 and *O. lucimarinus*). Our results showed that branch lengths are positively correlated with bootstrap values (Spearman’s rank correlation coefficient of 0.634, *p*-value < 2.2e-16) (Additional file 4: Figure S3B). Second, we examined 129 domain families with only one copy in each of the 9 green algal and 2 land plant species assuming that this group of domain families has not undergone gene gain and loss. Thus, their gene trees should be identical to the species tree in topology. Because the alternative scenario is possible (gene gains and losses have occurred) in these families, this is a conservative estimate of the orthology inference accuracy. Our results showed a varying degree of consistency in branching between gene trees and the species tree on different branches on the species tree, ranging from 37.2% to 98.3% (Additional file 5: Figure S4). Longer branches on the species tree, indicating greater evolutionary distance, correspond with higher consistency with gene trees. We should emphasize that in branches with high consistency, for example the branch leading to the split of *Micromonas* and *Ostreococcus* with 84% consistent trees, the gene loss to gene gain ratio was comparable to that in branches with low consistency, indicating that green algae have experienced extensive gene loss.

Several other observations are consistent with the extensive gene loss phenomenon we have inferred in green algal species. First, a substantial number of gene families were lost on lineages leading to *C. reinhardtii* and *O. lucimarinus* after the split from land plants [25]. Second, in *Ostreococcus*, downsizing of many gene families and gene losses were observed [26,27]. Third, a comparative analysis including *Micromonas* and *Ostreococcus* revealed that the common ancestor of Mamiellales had already experienced genome reduction [28]. Therefore, although lineage-specific gene gains took place, the lineage-specific losses contributed more significantly to the species-specific gene repertoire in green algae.

### Pseudogenes in green algae

**Figure 3:**
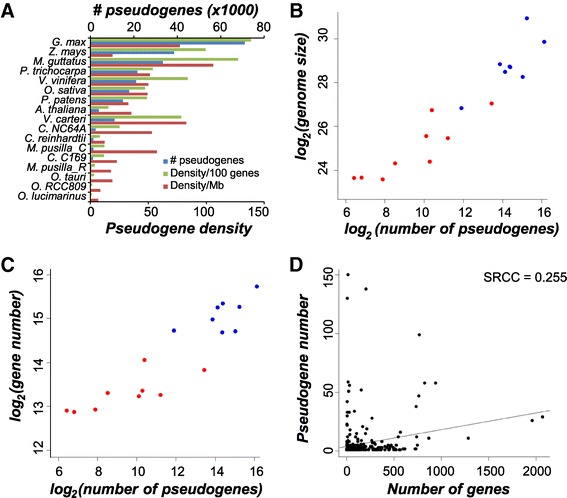
**Pseudogene counts in green algae and land plants. (A)**. Pseudogenes counts in total, per Mb genome, and per 100 genes in green algae and land plants. **(B)**. Number of pseudogenes and genome sizes in green algae (red) and land plants (blue). **(C)**. Number of pseudogenes and genes in green algae and plants. **(D)**. Number of genes and pseudogenes in each domain family in green algae. (SRCC: Spearman’s Rank Correlation Coefficient)

We found that genes are frequently lost throughout the green algal lineage. Some of the lost genes may still be present in the genome in the form of pseudogenes. Pseudogenes are defined as defunct genomic regions that are fragments of functional genes some with in-frame stops and/or frameshifts. To investigate pseudogenes in green algal genomes, we identified them using a modified pipeline [18]. A total of 18,352 pseudogenes were identified in all nine green algal species. In general, pseudogenes are less abundant in green algae than in land plants, even when normalized against genome sizes or total number of genes (Figure 3A). Among green algae and plants, the number of pseudogenes increases as the genome size and number of genes increase (Figure 3B and C, Pearson’s correlation coefficient of 0.95 and 0.91). Larger domain families also tend to have more pseudogenes in green algae (Figure 3D), although the correlation is weak (Spearman’s rank correlation coefficient of 0.26, *p*-value = 7.1e-07). This is possibly due to their small genome sizes and evolutionary pressure towards a more compact genome [26,27].

### Functions of retained *C. reinhardtii* genes duplicated after the *C. reinhardtii*-*V. carteri* split

Although gene losses appear to be more frequent, there are abundant retained duplicates throughout the green algal lineage. However, it is not clear if there is a functional bias among retained duplicates. To determine such a bias, we focused on *C. reinhardtii* since it is a green algal model organism with a relatively well-annotated gene set [29]. After annotating the *C. reinhardtii* proteome with GO categories based on sequence similarity (see Methods), 5,725 of 17,114 proteins (33.5%) are in ≥1 GO categories. During reconciliation of domain and species trees, the branches in the species tree where duplications took place were inferred as well. Thus we can examine functional biases of retained duplicates in each of the branches leading to *C. reinhardtii*. This information allows us to ask if functions of retained genes were consistent over the course of *C. reinhardtii* evolution. Focusing on the *C. reinhardtii* lineage after its split from the *V. carteri* lineage, 1,817 duplication events (involving 2,682 retained duplicates) took place in the *C. reinhardtii* lineage. Among 13 categories enriched in retained duplicates, they can be classified into the following three types.

**Figure 4:**
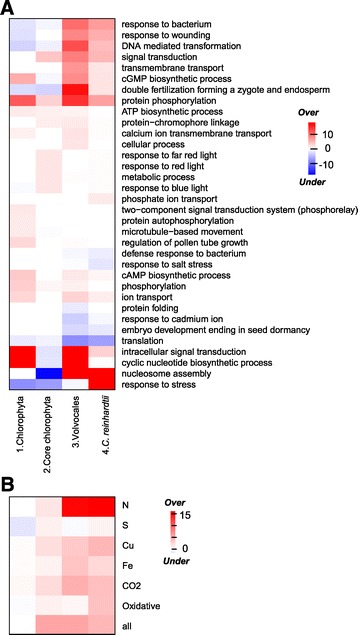
**Functions and stress responsiveness of retained duplicates in the** ***C. reinhardtii*** **lineage. (A)** Functions of retained duplicates. Columns indicate four phylogenetic branches as in Figure 2 with the most recent on the far right. Rows indicate GO biological processes terms with over or under-represented numbers of retained duplicate genes in at least one branch. Values shown are -log_10_(*p*) for over-represented and log_10_(*p*) for under-represented categories, respectively. **(B)** Stress responsiveness of retained duplicates. Stress conditions shown are N (nitrogen deprivation), S (sulfur deprivation), Cu (copper deprivation), Fe (iron deprivation), CO2 (CO_2_ deprivation), oxidative (oxidative stress), and all (all six conditions combined). Values shown are -log_10_(*p*) for over-representation and log_10_(*p*) for under-representation. Branches are the same as **(A)**.

The first type of functional categories belong to those involved in stress response (Figure 4A, Additional file 6: Table S4), similar to land plants and several other eukaryotes [11,12]. This result is further corroborated by the results from stress expression datasets (detailed in a later section), suggesting that these retained duplicates might have contributed to the species-specific stress response in green algae. One example is the heat shock protein Hsp20 family (PF00011): three duplication events took place in *C. reinhardtii* after the split from *V. carteri*, creating four *C. reinhardtii*-specific duplicates that are responsive to stress (at least one in six conditions mentioned below). The second type of retained duplicate enriched categories is transport including ion, phosphate, and transmembrane transport. One potential explanation is that functional divergence among duplicated transport genes allowed the regulation, affinity, and subcellular location of transporters to be fine tuned. For example, the potassium transporter family (PF02795) in *C. reinhardtii* experienced five duplication events after the split from *V. carteri* and resulted in six *C. reinhardtii*-specific duplicates, all of which are responsive to stress (at least one in six conditions mentioned below). These duplicates might have contributed to the species-specificity of ion transport and stress response in *C. reinhardtii*. In other green algal species, one example of such fine-tuning is the nitrate and ammonium transporters in *Micromonas* [30]; however, it is not limited to nutrient management: channelrhodopsins, ion-channels involved in light perception and phototaxis, are also found to be diverse even within the *Chlamydomonas* genus [31]. The third type of enriched categories is signaling. For example, nine retained duplicates are involved in the synthesis of cyclic nucleotides that are secondary messengers important for the regulation of flagella [32,33] and generally for the activation of ion channels [34]. Other examples of enriched signaling categories include protein phosphorylation and signal transduction (Figure 4A, Additional file 6: Table S4). Nucleosome assembly could also be involved in signaling as nucleosome is proposed to be a signaling module in addition to its function of DNA packaging [35]. Together, these enrichments indicate duplicates involved in complex environmental interactions and signaling systems tend to be retained, potentially because duplicates in these categories provide the capacity for responding to new environments and for new routes of regulation.

### Consistency of functional biases over the course of *C. reinhardtii* evolution

The above discussion was on the *C. reinhardtii* lineage after its split from the *V. carteri* lineage. In the process of green algal evolution, some duplicates may be preferentially retained throughout while others may be period-specific due to the ever-changing environment. In addition, different kinds of genes may have different longevities thus resulting in differences in enriched categories over time. To distinguish these possibilities, the timing of duplication of each retained gene was pinpointed to an internal or external branch that led to *C. reinhardtii*. The functions of ancestral genes at the time of duplication were assumed to be the same as those of their descendants. First we focus on categories that are branch specific. The branches are numbered as in Figures 2 and 4A. Stress response is enriched in just branch 4, while nucleosome assembly is enriched in 3 and 4. The expansion of stress related gene families in just the youngest branch may be because environmental condition would constantly change in the evolutionary history of the *C. reinhardtii* lineage. Thus previously retained duplicates conferring selective advantage may not be adaptive due to exposure of green algae to ever-changing environments.

In contrast to stress, we found that signaling categories are consistently enriched for retained duplicates over time. Further, retained duplicates in branch 3 and 4 tend to play markedly similar roles as branch 1, particularly those in ion transport, signaling and cyclic nucleotide synthesis. Retained duplicates in branch 2 were associated with several types of light response and signal transduction. (Figure 4A, Additional file 6: Table S4). This result suggests that gene families involved in signaling and to a lesser degree transport are constantly expanded in a lineage-specific manner, and more generally indicates consistent innovation in interaction with the environment. Further, these enrichments are consistent with other eukaryotes [11,12]. Three categories involved in double fertilization, pollen tube growth, and embryo development are not relevant to the single-cellular *C. reinhardtii*. They are likely annotated by the sequence similarity to plant proteins by Blast2go [36]. To test the robustness of our results, we tested the GO enrichment in the subset of domain families with ≥ 75% genes of a family only having one domain. The GO categories enriched in *C. reinhardtii* retained duplicates are similar to the results of all domain families (Additional file 7: Table S5). This illustrates that our results achieved using domain families instead of gene families are robust.

### Functional categories enriched in conserved genes and genes associated with pseudogenes

In addition to retained duplicates, we defined “conserved genes” as those with the same copy number (1 to 3) in green algal species. The categories enriched in conserved genes in *C. reinhardtii* were few and largely involved in housekeeping functions, including GO terms such as translation and two other ribosome-related terms. Other terms enriched in conserved genes included tetrapyrrole synthesis, and photosystem I reaction center (Additional file 8: Table S3). In addition to functional bias in retention and conservation, we would also like to examine functional bias in pseudogenization. To find out if there is such bias, we tested GO enrichment in genes associated with pseudogenes, defined as genes that are the closest relatives (by sequence similarity) to pseudogenized duplicates. Stress response was the only enriched biological process we found in genes associated with pseudogenes, indicating that, in addition to their high rate of retention, these genes have frequently undergone gene loss, a finding similar to a pseudogene study in *A. thaliana* [18]. This coupling between a high birth as well as high death rate is likely because genes involved in responding to specific stress conditions could become unnecessary when that condition does not persist.

### Retained duplicates and their stress responsiveness

Multiple stress conditions lead to increased oil content in microalgae [2]. In addition, we showed that stress response categories were enriched in retained duplicates in the *C. reinhardtii* lineage. However, GO annotation in *C. reinhardtii* is established solely based on computational approaches and has only 33.5% of genes annotated. To address these issues, we asked if retained genes tend to be stress responsive compared to singletons under six conditions: deficiency in N, sulfur, iron, copper, and CO2, and oxidative stress [37-42]. In all stress conditions except sulfur deprivation, stress responsiveness tends to be over-represented among retained genes (p-value ≤0.01), (Figure 4B, Additional file 9: Table S2). When combining all six RNA-seq datasets and defining stress responsiveness of a gene as being responsive in ≥1 conditions, retained duplicates still tend to be stress responsive (Figure 4B, Additional file 9: Table S2). This result corroborates our conclusion that retained duplicates tend to be in the stress response functional categories.

According to functional category analysis, stress response was not enriched among retained duplicates except in branch 4, which is the most recent branch (Figure 4A). Consistent with this finding, analysis of stress expression data also revealed that retained genes tend to be stress responsive in more conditions if they were duplicated more recently (Figure 4B), with 5 conditions in branch 4 and gradually declining to none in branch 1. This is also consistent with our finding that the closest relatives of pseudogenes tend to be involved in stress response, again reinforcing the idea that, although stress response genes tend to be retained at a higher rate compared to genome average, they tend to be more short-lived.

### Stress response evolution post duplication in the *C. reinhardtii lineage*

Retained duplicates tend to be stress responsive (Figure 4A and B), suggesting that gene duplication might provide a source for innovations in response to stress. This possibility then leads to the question how often innovation, defined as gain of stress responsiveness from a non-responsive ancestral gene, occurred in green algae. To answer this question, we first integrated phylogenetic data and stress related expression datasets to infer the ancestral response state (Additional file 2: Figure S1C). With ancestral states, the gain and loss events of duplicated genes can then be distinguished [19]. Stress response states are defined as U (up-regulated by ≥2 fold, false discovery rate ≤ 5%), D (down-regulated by ≥2 fold, false discovery rate ≤ 5%), and N (not significantly changed). Only the ancestral nodes leading immediately to extant genes were included in our analysis to avoid complications from predicting responses in nested branches [19].

**Figure 5:**
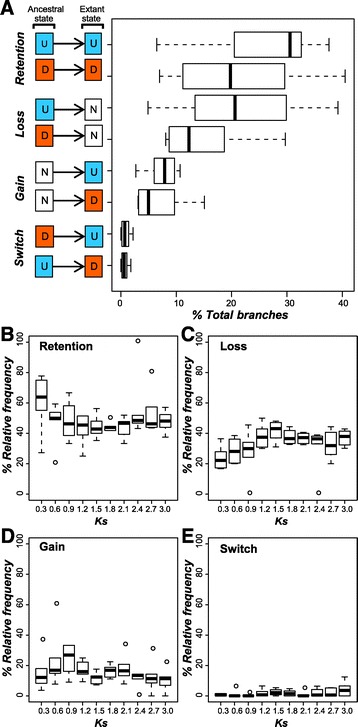
**Stress response evolution scenarios of** ***C. reinhardtii*** **duplicates compared to their ancestral genes. (A)**. There are four scenarios as indicated on the far left column. The ancestral state column shows the reconstructed ancestral response state and the extant state column shows the extant gene response state. ‘U’, ‘D’, and ‘N’ indicate up-regulated, down-regulated, and not-regulated under a stress condition, respectively. On the right, the preponderance (in percent total) of each scenario for a given stress condition is shown. The percent of each scenario for six conditions are summarized in a boxplot for each ancestral-extant combination. **(B-E)**. Stress response evolution among *C. reinhardtii* paralogs using Ks as a proxy for time. X-axis indicates Ks values, and Y-axis indicates the relative frequency of each stress evolution scenario. **(B)**. stress response retention. **(C)**. loss. **(D)**. gain. **(E)**. switch.

We define four “evolutionary events” (retention, gain, loss, or switch) based on a comparison of stress response states between an extant gene and its most immediate ancestral gene node for each of the six stress conditions. We found that 6,330 comparisons (10.9%) were relevant because they involved either extant and/or ancestral stress responsive genes. Among the six stress conditions, the median number of events involving retention of the ancestral stress response is 48% (32% U - > U, and 16% D - > D). We also found that 35% were response loss events (23% U - > N, and 12% D - > N). Meanwhile, comparatively fewer events (13%) involved functional gain (9% N - > U, and 4% N - > D) and even fewer events (2%) involved functional switch (1% D - > U, and 1% U - > D) (Figure 5A). To find out if younger duplicates tend to retain their ancestral response state, we analyzed the relative frequencies of all four stress response evolution scenarios against time, using synonymous substitution rate (Ks) as a proxy for time (Figure 5B-E). Regardless of the Ks value, it is generally true that the rates of stress response evolution scenarios are retention > loss > gain > > switch. However, the relative abundance of each scenario changed over time (Figure 5B-E). Rate of retention in bins with smaller Ks (0.3 and 0.6) is higher than in bins with larger Ks (Wilcoxon rank sum test: W = 413, p-value = 0.021). When 0 ≤ Ks ≤ 0.9, the rate of retention decreased from 63.8% to 48.2% (median of all conditions), and it remains relatively stable afterwards (Figure 5B). On the contrary, rate of loss in bins with smaller Ks (0.3, 0.6, and 0.9) is lower than in bins with larger Ks (Wilcoxon rank sum test: W = 156, p-value = 3.5e-4). The rate of loss increased from 22.2% to 37.5% when 0 ≤ Ks ≤ 1.2, and remains relatively stable after that (Figure 5C). These results show that younger duplicate genes tend to retain the ancestral stress response state, similar to *A. thaliana* [19]. The rate of functional gain peaked at Ks = 0.9, and decreased thereafter, as similar pattern was observed in *A. thaliana* [19].

Nonetheless, compared to the results of a similar study in *A. thaliana* [19], *C. reinhardtii* has comparable rates in response retention, loss, and switch, but a much higher rate of functional gain (13% versus 6%). Note that we examined only six abiotic conditions in *C. reinhardtii* compared to the 16 conditions encompassing biotic and abiotic stress environments in the *A. thaliana* study [19]. Considering that the relative abundance of the evolutionary scenarios is similar across divergent conditions [19], having more data will likely not contribute to significant changes in the gain rate estimate in either direction. We should also emphasize that, regardless of the Ks value, *C. reinhardtii* consistently has a higher rate of functional gain (Figure 5D) when compared to *A. thaliana* [19]. Taken together, our findings indicate that innovation occurs more often in *C. reinhardtii* than in *A. thaliana* in the context of stress response. The excess gain events could be due to the fact that *C. reinhardtii* is a single cellular organism and has a shorter life cycle while encountering more diverse environmental conditions, as one would expect that a shorter life cycle would lead to a higher number of mutations per unit time compared to species with a longer generation time, thus providing more raw material for adaptation to occur upon.

## Conclusions

Our analysis of gene family evolution, functional evolution and pseudogenization in the green algal lineage complement previous studies in other eukaryotes, reinforcing that the association between lineage-specific evolution and stress response is a common feature of eukaryotes. This association is likely due to the selective pressure under ever-changing environment. In this scenario, stress gene duplicates were frequently under positive selection. In addition, the high rate of innovation in acquiring abilities to respond to stress in *C. reinhardtii* duplicates contributes to a highly diverse stress responsive gene repertoire that can potentially be adaptive.

The model organism *C. reinhardtii* is used to study the mechanism of stress response in green algae although it is not a direct candidate for biofuel production [37]. As the general metabolic changes under stress might be similar across divergent micro-algal species [43], the particular genes involved in stress response could be quite different as they are shaped by lineage-specific family expansion and subsequent gain-of-function events. Such species-specificity cannot be deciphered when focusing on one model organism. Therefore, in addition to focusing on the well-established model organism of *C. reinhardtii* to discover the general biology of stress response in green algae, it is necessary to investigate diverse green algal species to discover their uniqueness in stress response, towards the ultimate goal of finding the perfect alga for biofuel production.

## Methods

### Protein domains in green algal species

Genome and protein sequences of nine green algal species (see Background) and eight land plant species were obtained from the US Department of Energy Joint Genome Institute (www.jgi.doe.gov) and Phytozome (www.phytozome.net, version 7.0). The nine green algal species included are *Micromonas pusilla* RCC299 [28], *Micromonas pusilla* CCMP1545 [28], *Ostreococcus* sp.RCC809 (www.jgi.doe.gov, unpublished, restrictions of publishing lifted), *Ostreococcus tauri* [26], *Ostreococcus lucimarinus* [27], *Chlorella* NC64A [44], *Coccomyxa* sp.C169 [20], *C. reinhardtii* [29], and *Volvox carteri* [45]. The land plant species are *Populus trichocarpa* [46], *Glycine max* [47], *A. thaliana* [48], *Vitis vinifera* [49], *Mimulus guttatus* [50], *Zea mays* [51], *Oryza sativa* [52], and *P. patens* [53]. Some of these sequence data were produced by the DOE JGI in collaboration with the user community. HMMER [54] was used with trusted cutoff to scan algal and plant protein sequences for Pfam domains [21]. Fisher’s exact test was used to test the enrichment of GO categories in conserved domain families in green algae and expanded domain families in green algae using GO annotation on Pfam domains (ftp://ftp.ebi.ac.uk/).

To assess the completeness of gene annotation in green algae, we identified intergenic regions with coding potential and domain presence. Green algal protein sequences were aligned to green algal genome sequences with BLAST (tblastn, E-value threshold ≤ 1e-5, [55]). Matches with ≥30 amino acids long and ≥40% identity were kept and the matching genomic sequences were translated into peptide sequences using Genewise [56]. After filtering out sequences with frameshifts and identical sequences, the rest were consolidated by concatenating sequences with ≥5 amino acid overlaps. Protein domains in the concatenated sequences were identified with HMMER using trusted cutoff. Domains overlapping with domains identified in annotated genes or pseudogenes (see later section) were eliminated. Overlapping domain sequences were merged and identical sequences removed (Additional file 2: Figure S1A).

### Identification of missing domains in green algal genomes with variable annotation qualities

The robustness of our domain family analysis is fundamentally dependent on the quality of gene annotation in green algae. For example, an un-annotated domain would lead to a false prediction of gene loss. Most of the green algal genome annotations are automatically generated using computational approaches with various degrees of manual intervention. Thus, the quality of the annotation is likely highly variable and some genes and thus protein domains may not be annotated. Given our goal is to evaluate the gain and loss patterns of algal domain families, these potentially missing domains are false negatives that can have a significant impact on our subsequent studies. Thus, to identify domains in the genomes that are missed by current annotation, we aligned all the protein sequences of nine green algal species to their genomes to identify all sequences with coding potential.

A total of 810,578 matches were identified. The matching genomic sequences were translated into peptide sequences using Genewise [56]. After removing redundant sequences that were identical in their entirety and merging overlapping sequences with identical amino acids in the overlapping region, 474,350 sequences remained. Overlapping sequences that were not identical in the overlapping regions were not removed since they might contain different domains. We identified 238,446 domains from these non-redundant sequences. After removing identical domain sequences, merging overlapping domain sequences, removing domain sequences overlapping with domains in annotated genes and pseudogenes identified using a published pipeline [18], 6,432 domain sequences remained and are referred to as domains in un-annotated regions, of which one-third are in *V. Carteri* (Additional file 10: Figure S2B). They are likely to be domains residing partially or completely in intronic or intergenic regions. Out of the 1,985 *V. carteri* domains, 862 are retrotransposon related, while a total of 223 domains in all eight other species are retrotransposon related. Most of the domains in un-annotated regions are shorter than the average of annotated domains (Additional file 10: Figure S2A), and the number is very small compared to the annotated domains in each species (Additional file 10: Figure S2B). For these reasons, we conclude that despite the automated nature of the current green algal annotations they contain most of the known Pfam domains in green algae and further analysis was done only using the annotated domains.

### Defining green algal orthologous groups (OGs) and lineage-specific expansions (LSEs)

To build a phylogeny for each protein domain X, sequences of domain X were extracted from full length protein sequences of the green algae and land plants and aligned using MAFFT [57]. Using the alignments generated, the phylogeny of each domain family was inferred using RAxML [58] with parameters -f d -m PROTGAMMAJTT. Domain family trees were reconciled with a previously published species tree [24] using NOTUNG [59]. Notung compares a gene tree to a species tree to infer whether a node in a gene tree leads to bifurcating branches due to speciation or duplication. From the speciation and duplication node classifications, we inferred orthologous and paralogous relationships as well as gene gain and loss events for further analyses. We have also used the 18 s rRNA sequences of two land plants and all nine green algal species to built a species phylogeny using RAxML [58] with parameters -f a -x 12345 -p 12345 -# 1000 -m GTRGAMMA. Given the topology is highly similar, only the previously published phylogeny was used. For large domain families that RAxML run didn’t finish in 160 hours, a neighbor joining tree was built with PHYLIP [60] and the distance trees were broken down to smaller sub-clusters with ≥ 4 genes and ≤ 300 genes, and distance to root ≥ 0.05. Each sub-cluster was regarded as a “sub-family”. Domain sequences of sub-family members were used to identify orthologous groups with the same approach as above (Additional file 2: Figure S1B). To test the robustness of our orthology inference, bootstrap values were acquired on 1,500 randomly chosen domain families using RAxML (−f a -# 400 -m PROTGAMMAJTT -x 12345). Bootstrap values and branch length are plotted on Additional file 4: Figure S3B.

To functionally annotate the *C. reinhardtii* genome, protein sequences were first aligned to the nr protein database (ftp://ftp.ncbi.nlm.nih.gov/blast/db/) to identify putative homologs with an e-value cut-off of 1e-5. GO annotation was then inferred using blast2go based on genes with GO entries in the nr database [36]. Fisher’s exact test was used to test the enrichment of retained duplicates in GO categories and a false discovery rate [61] adjusted p-value threshold of 0.05 was used.

### Pseudogenes in green algae

Pseudogenes of green algae were predicted using a previously defined pipeline [18] with some modifications: 1) we used the whole genomes for the BLAST search and filtered out hits overlapping with genes as opposed to using the intergenic sequences only; 2) we made the pseudoexon merging recursive so that multiple pseudogenes can be derived from one pseudoexon cluster provided that the merged pseudogenes do not overlap with each other; 3) RepeatMasker (ver. 3.3.0) was run after the pseudogene pipeline on pseudogene sequences using Viridiplantae repeats (Cutoff = 300, Divergence = 30) and pseudogenes with hits other than “Simple_repeat”, “Low_complexity” and “Satellite” were removed from the dataset; 4) to control for false positives due to proteins being split between contigs, we removed pseudogenes that were within the 95^th^ percentile genomic intron length from the end of a contig if they do not have disabling mutations, defined as a frame shift or premature stop codon. A “high confidence pseudogene” has one or more disabling mutations and has the following conditions satisfied: the distance between the pseudogene and the ends of the contig larger than the distance between the matching region and the end of the protein plus 95^th^ percentile genomic intron length on both ends.

### Inferring ancestral stress response state

To identify genes responsive to various stress conditions, *C. reinhardtii* RNA-seq datasets of N [37], S [38], Fe [39], Cu [40], and CO2 deficiency [41], and oxidative stress [42], were obtained from the Sequence Read Archive at NCBI (http://www.ncbi.nlm.nih.gov/sra). Reads were aligned to *C. reinhardtii* genome using Tophat [62], with following options: −i 13 -I 8712 -g 1. For each dataset, differential expression was determined using EdgeR [63] with a threshold of fold change ≥ 2 and false discovery rate ≤ 5%. Domain family phylogenies and extant stress response states were combined to infer the ancestral stress response states of *C. reinhardtii* genes using BayesTraits [64]. The ancestral response state was inferred with maximum likelihood under the assumption that the probability of response change is proportional to the branch length on the domain family phylogeny. Three discrete functional states were defined as 1) up-regulation (u, by ≥ 2 fold and FDR ≤ 0.05), 2) down-regulation (d, by ≥ 2 fold and FDR ≤ 0.05), and 3) no-regulation (n). The ancestral states were estimated using multistate, maximum likelihood and most recent common ancestor (MRCA) options for each family phylogeny and each stress condition. Only ancestral states with a posterior probability > 0.5 were used for subsequent analysis. BayesTraits cannot be used in cases that all genes in one tree had same state, and we assumed in that case all ancestral genes had the same state as the extant ones. Ancestral gene response states were compared to extant gene response states to infer innovations and losses in stress response (Additional file 2: Figure S1C).

## Additional files

Additional file 1: Table S1. Gene and Pfam domain counts in green algae and land plants. Additional file 2: Figure S1. Analysis pipelines. (A). Pipeline used to identify missing domains. (B). Pipeline for identifying retained duplicates in green algal lineage. (C). Pipeline for ancestral stress response state inference in *C. reinhardtii*. Additional file 3: Figure S5. Green algal species tree based on 18 s rRNA sequences. Numbers indicate the bootstrap values. Additional file 4: Figure S3. Bootstrap values of various branches. (A). Bootstrap value distribution of all branches in phylogenetic trees of 1,500 domain families. (B). Distributions of bootstrap values in the domain phylogenetic trees (X-axis) and branch length (Y-axis). Additional file 5: Figure S4. Consistency between domain and species tree. The species tree topology is shown. The number on each branch indicates the percentage of domain family trees with the same branching pattern as the species tree on the branch in question. Additional file 6: Table S4. Gene Ontology biological processes significantly enriched in *C. reinhardtii* retained duplicates. Additional file 7: Table S5. Gene Ontology biological processes significantly enriched in *C. reinhardtii* retained duplicates in a subset of domain families. Additional file 8: Table S3. Gene Ontology categories significantly enriched in conserved genes or genes most closely related to pseudogenes in *C. reinhardtii*. Additional file 9: Table S2.
*C. reinhardtii* retained duplicate genes tend to be stress responsive. Additional file 10: Figure S2. Distribution of domains in un-annotated regions (DURs) in green algae. (A). DUR distribution by length. X-axis shows the length of DURs as fraction of the average length of annotated domains in the same family. Y-axis indicates frequency. (B). Preponderance of DURs among green algal species. Blue: the number of DURs in each green algal species. Red: the number of DURs relative to the number of annotated domains. Green: the number of DURs that are longer than half of the average lengths of annotated domains in the same family relative to the number of annotated domains in each species.
