# Supplementary material for: Retained duplicate genes in green alga Chlamydomonas reinhardtii tend to be stress responsive and experience frequent response gains
Source: BMC Genomics. 2015 Mar 4;16(1):149. doi: 10.1186/s12864-015-1335-5 (PMC4364661; doi:10.1186/s12864-015-1335-5)
Supplement: Additional file 1: Table S1. — Gene and Pfam domain counts in green algae and land plants. [file 12864_2015_1335_MOESM1_ESM.doc]

Supplemental Table 1. Gene and Pfam domain counts in green algae and land plants

|  | **total genes** | **genes with domain** | **fraction** |
| --- | --- | --- | --- |
| ***M. pusilla_*R** | 10109 | 7038 | 0.70 |
| ***M. pusilla_*C** | 10547 | 6837 | 0.65 |
| ***O.* RCC809** | 7492 | 5366 | 0.72 |
| ***O. tauri*** | 7725 | 5253 | 0.68 |
| ***O. lucimarinus*** | 7651 | 5681 | 0.74 |
| ***C.* NC64A** | 9791 | 7411 | 0.76 |
| ***C.* C169** | 9629 | 6615 | 0.69 |
| ***V. carteri*** | 14542 | 8198 | 0.56 |
| ***C. reinhardtii*** | 17114 | 9736 | 0.57 |
| ***S. moellendorffii*** | 22285 | 15824 | 0.71 |
| ***P. patens*** | 32273 | 17509 | 0.54 |
| ***O. sativa*** | 55802 | 35531 | 0.64 |
| ***Z. mays*** | 39656 | 25984 | 0.66 |
| ***M. guttatus*** | 28282 | 23136 | 0.82 |
| ***V. vinifera*** | 26346 | 18841 | 0.72 |
| ***A. thaliana*** | 27416 | 21749 | 0.79 |
| ***G. max*** | 46367 | 38774 | 0.84 |
| ***P. trichocarpa*** | 40668 | 30231 | 0.74 |
