# Supplementary figures and images for: Retained duplicate genes in green alga Chlamydomonas reinhardtii tend to be stress responsive and experience frequent response gains
Source: BMC Genomics. 2015 Mar 4;16(1):149. doi: 10.1186/s12864-015-1335-5 (PMC4364661; doi:10.1186/s12864-015-1335-5)

## Supplemental Figure 1

**A**

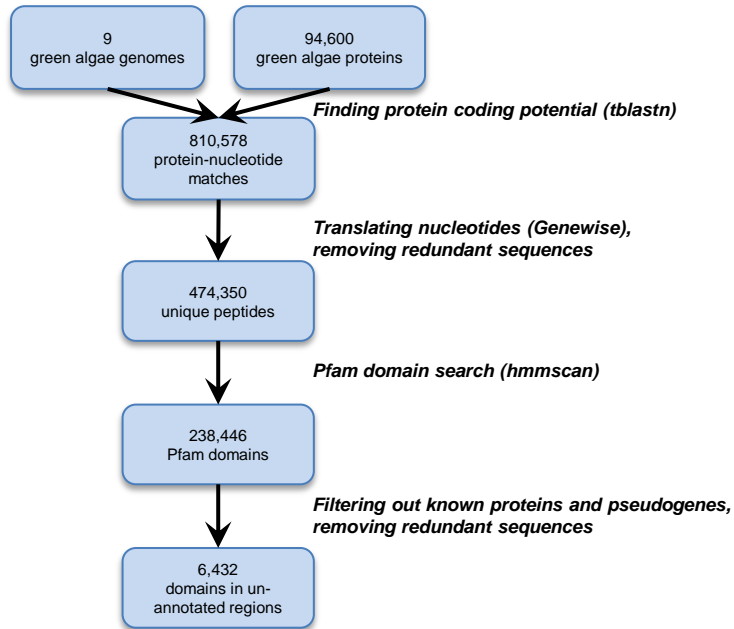

**B**

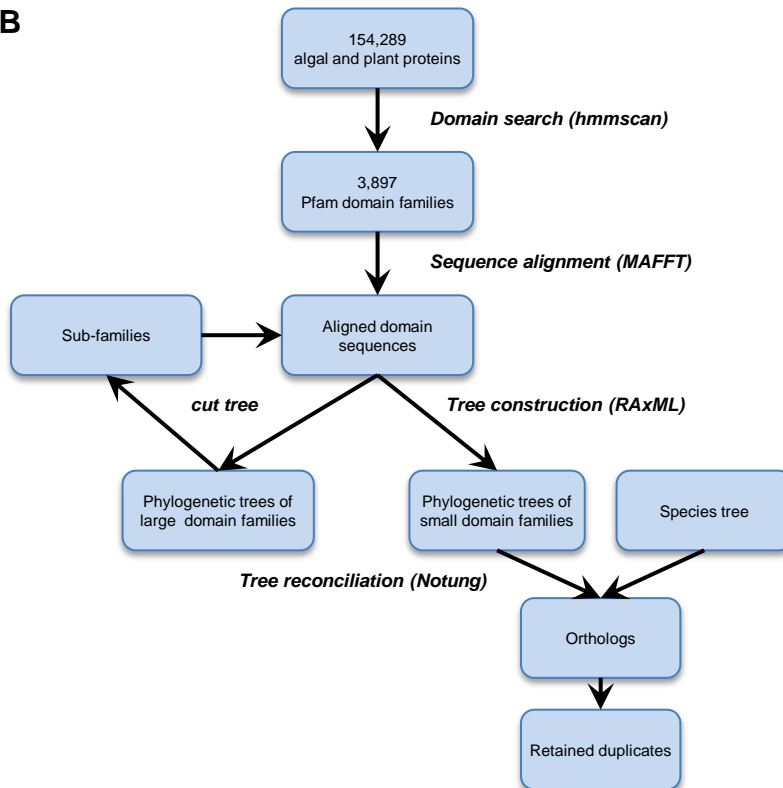

**C**

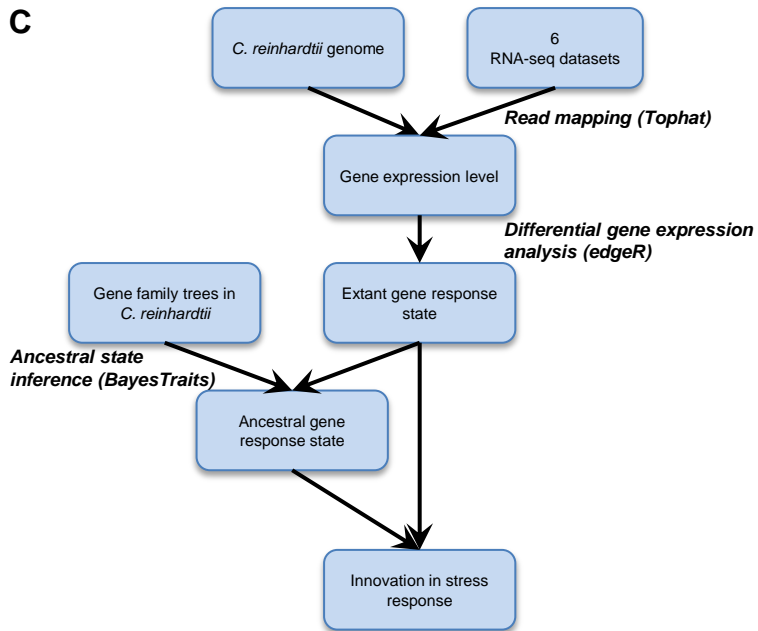

Supplement: Additional file 2: Figure S1. — Analysis pipelines. (A). Pipeline used to identify missing domains. (B). Pipeline for identifying retained duplicates in green algal lineage. (C). Pipeline for ancestral stress response state inference in C. reinhardtii. [file 12864_2015_1335_MOESM2_ESM.pdf]

## Supplemental Figure 5

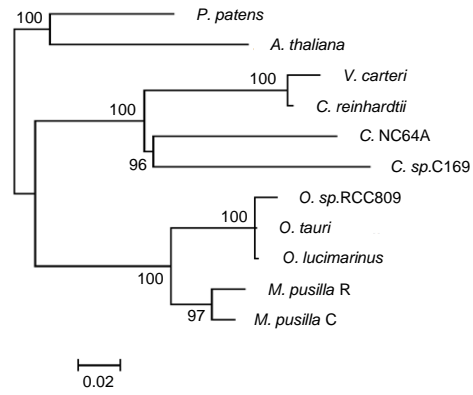

Supplement: Additional file 3: Figure S5. — Green algal species tree based on 18 s rRNA sequences. Numbers indicate the bootstrap values. [file 12864_2015_1335_MOESM3_ESM.pdf]

Supplemental Figure 3

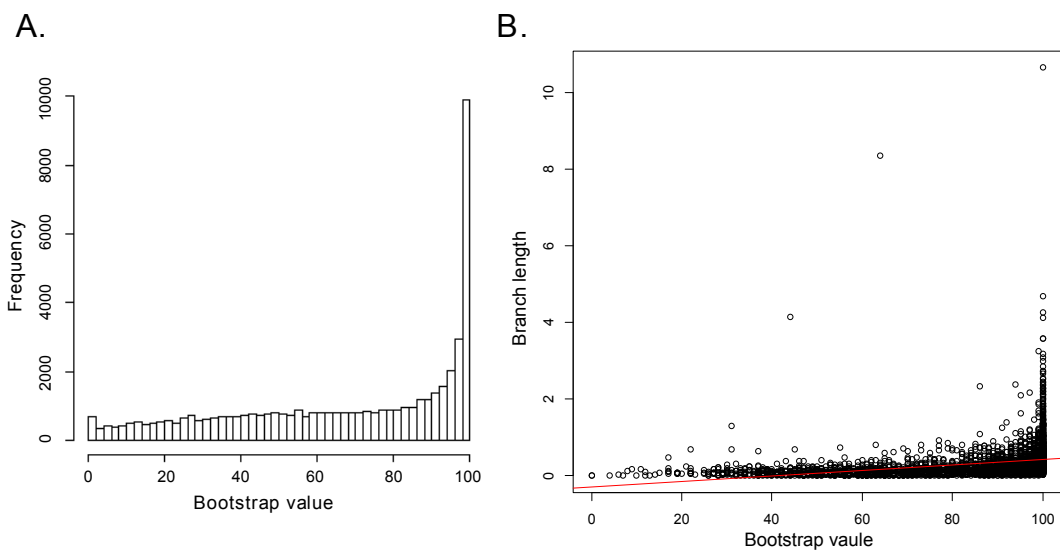

Supplement: Additional file 4: Figure S3. — Bootstrap values of various branches. (A). Bootstrap value distribution of all branches in phylogenetic trees of 1,500 domain families. (B). Distributions of bootstrap values in the domain phylogenetic trees (X-axis) and branch length (Y-axis). [file 12864_2015_1335_MOESM4_ESM.pdf]

Supplemental Figure 4

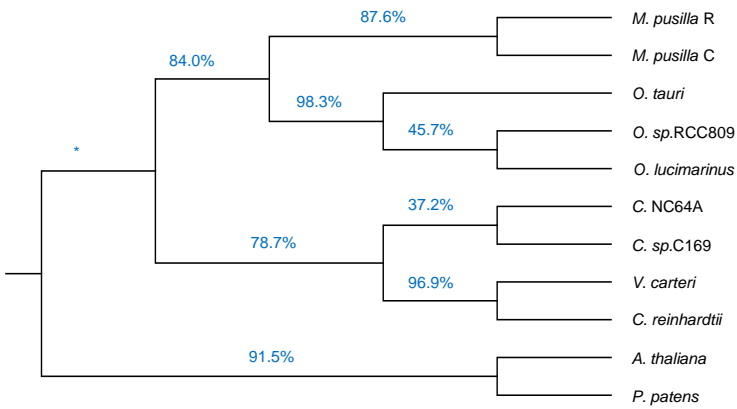

\*: not enough families to estimate percentage

Supplement: Additional file 5: Figure S4. — Consistency between domain and species tree. The species tree topology is shown. The number on each branch indicates the percentage of domain family trees with the same branching pattern as the species tree on the branch in question. [file 12864_2015_1335_MOESM5_ESM.pdf]

Supplemental Figure 2

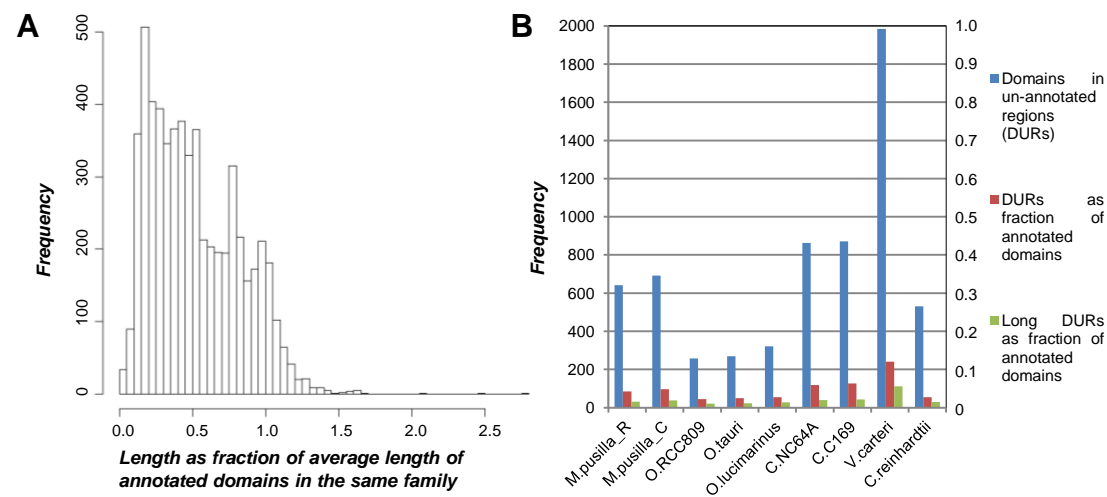

Supplement: Additional file 10: Figure S2. — Distribution of domains in un-annotated regions (DURs) in green algae. (A). DUR distribution by length. X-axis shows the length of DURs as fraction of the average length of annotated domains in the same family. Y-axis indicates frequency. (B). Preponderance of DURs among green algal species. Blue: the number of DURs in each green algal species. Red: the number of DURs relative to the number of annotated domains. Green: the number of DURs that are longer than half of the average lengths of annotated domains in the same family relative to the number of annotated domains in each species. [file 12864_2015_1335_MOESM10_ESM.pdf]
