# Supplementary material for: Retained duplicate genes in green alga Chlamydomonas reinhardtii tend to be stress responsive and experience frequent response gains
Source: BMC Genomics. 2015 Mar 4;16(1):149. doi: 10.1186/s12864-015-1335-5 (PMC4364661; doi:10.1186/s12864-015-1335-5)
Supplement: Additional file 6: Table S4. — Gene Ontology biological processes significantly enriched in C. reinhardtii retained duplicates. [file 12864_2015_1335_MOESM6_ESM.doc]

**Supplemental Table 4.** Gene Ontology biological processes significantly enriched in *C. reinhardtii* retained duplicates

| **GO** | **Annotation** | **GOD^a^** | **GON^b^** | **NGOD^c^** | **NGON^d^** | ***p*^e^** | **FDR^f^** |
| --- | --- | --- | --- | --- | --- | --- | --- |
| *C. reinhardtii* lineage (4) | |  |  |  |  |  |  |
| GO:0006950 | response to stress | 109 | 31 | 770 | 4815 | 6.23E-63 | 2.25E-59 |
| GO:0006334 | nucleosome assembly | 94 | 32 | 785 | 4814 | 2.80E-51 | 3.38E-48 |
| GO:0009617 | response to bacterium | 23 | 15 | 856 | 4831 | 2.26E-10 | 1.02E-07 |
| GO:0006468 | protein phosphorylation | 57 | 108 | 822 | 4738 | 4.97E-10 | 1.80E-07 |
| GO:0009611 | response to wounding | 22 | 18 | 857 | 4828 | 6.96E-09 | 1.94E-06 |
| GO:0007165 | signal transduction | 27 | 33 | 852 | 4813 | 3.94E-08 | 1.02E-05 |
| GO:0009294 | DNA mediated transformation | 20 | 18 | 859 | 4828 | 9.18E-08 | 2.08E-05 |
| GO:0035556 | intracellular signal transduction | 25 | 39 | 854 | 4807 | 3.19E-06 | 5.24E-04 |
| GO:0009567 | double fertilization forming a zygote and endosperm | 15 | 19 | 864 | 4827 | 5.78E-05 | 8.71E-03 |
| GO:0006182 | cGMP biosynthetic process | 9 | 6 | 870 | 4840 | 9.50E-05 | 1.23E-02 |
| GO:0055085 | transmembrane transport | 28 | 61 | 851 | 4785 | 1.41E-04 | 1.70E-02 |
| GO:0006817 | phosphate ion transport | 6 | 2 | 873 | 4844 | 2.73E-04 | 2.99E-02 |
| GO:0006811 | ion transport | 10 | 11 | 869 | 4835 | 4.84E-04 | 4.61E-02 |
| Volvocales lineage (3) | |  |  |  |  |  |  |
| GO:0006334 | nucleosome assembly | 90 | 36 | 1094 | 4505 | 3.09E-35 | 5.58E-32 |
| GO:0035556 | intracellular signal transduction | 52 | 12 | 1132 | 4529 | 2.50E-25 | 3.01E-22 |
| GO:0009190 | cyclic nucleotide biosynthetic process | 41 | 4 | 1143 | 4537 | 3.09E-24 | 2.24E-21 |
| GO:0009567 | double fertilization forming a zygote and endosperm | 32 | 2 | 1152 | 4539 | 3.25E-20 | 1.31E-17 |
| GO:0006468 | protein phosphorylation | 83 | 82 | 1101 | 4459 | 9.12E-18 | 3.00E-15 |
| GO:0009294 | DNA mediated transformation | 31 | 7 | 1153 | 4534 | 1.21E-15 | 3.13E-13 |
| GO:0009617 | response to bacterium | 29 | 9 | 1155 | 4532 | 2.47E-13 | 5.25E-11 |
| GO:0007165 | signal transduction | 37 | 23 | 1147 | 4518 | 4.64E-12 | 8.82E-10 |
| GO:0009611 | response to wounding | 28 | 12 | 1156 | 4529 | 2.17E-11 | 3.74E-09 |
| GO:0006182 | cGMP biosynthetic process | 15 | 0 | 1169 | 4541 | 5.05E-11 | 7.94E-09 |
| GO:0055085 | transmembrane transport | 45 | 44 | 1139 | 4497 | 2.80E-10 | 4.05E-08 |
| GO:0006811 | ion transport | 14 | 7 | 1170 | 4534 | 6.51E-06 | 6.93E-04 |
| GO:0070588 | calcium ion transmembrane transport | 7 | 1 | 1177 | 4540 | 1.05E-04 | 8.34E-03 |
| GO:0009987 | cellular process | 52 | 103 | 1132 | 4438 | 1.70E-04 | 1.31E-02 |
| GO:0006171 | cAMP biosynthetic process | 8 | 3 | 1176 | 4538 | 2.96E-04 | 2.19E-02 |
| GO:0018298 | protein-chromophore linkage | 6 | 1 | 1178 | 4540 | 4.46E-04 | 2.94E-02 |
| Core Chlorophyta lineage (2) | |  |  |  |  |  |  |
| GO:0007165 | signal transduction | 39 | 21 | 1792 | 3873 | 1.69E-07 | 8.74E-05 |
| GO:0006468 | protein phosphorylation | 83 | 82 | 1748 | 3812 | 1.01E-06 | 4.56E-04 |
| GO:0008152 | metabolic process | 86 | 102 | 1745 | 3792 | 6.40E-05 | 1.35E-02 |
| GO:0009637 | response to blue light | 13 | 3 | 1818 | 3891 | 6.98E-05 | 1.35E-02 |
| GO:0010218 | response to far red light | 14 | 4 | 1817 | 3890 | 8.51E-05 | 1.40E-02 |
| GO:0010114 | response to red light | 15 | 5 | 1816 | 3889 | 9.58E-05 | 1.44E-02 |
| GO:0018298 | protein-chromophore linkage | 7 | 0 | 1824 | 3894 | 3.40E-04 | 4.55E-02 |
| Chlorophyta lineage (1) | |  |  |  |  |  |  |
| GO:0035556 | intracellular signal transduction | 61 | 3 | 1538 | 4123 | 1.15E-30 | 4.16E-27 |
| GO:0009190 | cyclic nucleotide biosynthetic process | 44 | 1 | 1555 | 4125 | 9.07E-24 | 1.09E-20 |
| GO:0006468 | protein phosphorylation | 94 | 71 | 1505 | 4055 | 3.48E-15 | 2.10E-12 |
| GO:0006182 | cGMP biosynthetic process | 15 | 0 | 1584 | 4126 | 4.68E-09 | 1.41E-06 |
| GO:0006171 | cAMP biosynthetic process | 11 | 0 | 1588 | 4126 | 7.87E-07 | 1.90E-04 |
| GO:0016310 | phosphorylation | 59 | 63 | 1540 | 4063 | 1.76E-06 | 3.98E-04 |
| GO:0006811 | ion transport | 16 | 5 | 1583 | 4121 | 5.85E-06 | 1.11E-03 |
| GO:0080092 | regulation of pollen tube growth | 9 | 0 | 1590 | 4126 | 1.02E-05 | 1.75E-03 |
| GO:0000160 | two-component signal transduction system (phosphorelay) | 8 | 1 | 1591 | 4125 | 2.48E-04 | 2.89E-02 |
| GO:0006754 | ATP biosynthetic process | 9 | 2 | 1590 | 4124 | 3.15E-04 | 3.56E-02 |
| GO:0046777 | protein autophosphorylation | 11 | 4 | 1588 | 4122 | 3.33E-04 | 3.61E-02 |
| GO:0007018 | microtubule-based movement | 19 | 14 | 1580 | 4112 | 3.44E-04 | 3.61E-02 |

^a^GOD indicates number of retained duplicates with GO. ^b^GON, number of genes that are not retained duplicates with GO. ^c^NGOD, number of retained duplicates without GO. ^d^NGON, number of genes that are not retained duplicates without GO. ^e^p value is calculated using Fisher’s exact test. ^f^FDR value is calculated using R package qvalue. Numbers in parenthesis indicate branches as shown on Figure 2.
