# Supplementary material for: Retained duplicate genes in green alga Chlamydomonas reinhardtii tend to be stress responsive and experience frequent response gains
Source: BMC Genomics. 2015 Mar 4;16(1):149. doi: 10.1186/s12864-015-1335-5 (PMC4364661; doi:10.1186/s12864-015-1335-5)
Supplement: Additional file 7: Table S5. — Gene Ontology biological processes significantly enriched in C. reinhardtii retained duplicates in a subset of domain families. [file 12864_2015_1335_MOESM7_ESM.doc]

**Supplemental Table 5.** Gene Ontology biological processes significantly enriched in *C. reinhardtii* retained duplicates in a subset of domain families

| **GO** | **Annotation** | **GOD^a^** | **GON^b^** | **NGOD^c^** | **NGON^d^** | ***p*^e^** | **FDR^f^** |
| --- | --- | --- | --- | --- | --- | --- | --- |
| *C. reinhardtii* lineage (4) | |  |  |  |  |  |  |
| GO:0006950 | response to stress | 109 | 20 | 450 | 2855 | 6.51E-69 | 1.70E-65 |
| GO:0006334 | nucleosome assembly | 94 | 32 | 465 | 2843 | 4.96E-50 | 4.32E-47 |
| GO:0009617 | response to bacterium | 22 | 12 | 537 | 2863 | 2.47E-10 | 8.05E-08 |
| GO:0009611 | response to wounding | 22 | 12 | 537 | 2863 | 2.47E-10 | 8.05E-08 |
| GO:0007165 | signal transduction | 25 | 24 | 534 | 2851 | 1.55E-08 | 3.38E-06 |
| GO:0009294 | DNA mediated transformation | 20 | 16 | 539 | 2859 | 6.92E-08 | 1.39E-05 |
| GO:0035556 | intracellular signal transduction | 25 | 38 | 534 | 2837 | 6.13E-06 | 1.00E-03 |
| GO:0009567 | double fertilization forming a zygote and endosperm | 15 | 19 | 544 | 2856 | 1.12E-04 | 1.40E-02 |
| GO:0006182 | cGMP biosynthetic process | 9 | 6 | 550 | 2869 | 1.50E-04 | 1.63E-02 |
| GO:0006817 | phosphate ion transport | 6 | 2 | 553 | 2873 | 3.78E-04 | 3.41E-02 |
| GO:0006811 | ion transport | 7 | 4 | 552 | 2871 | 5.29E-04 | 4.60E-02 |
| Volvocales lineage (3) | |  |  |  |  |  |  |
| GO:0006334 | nucleosome assembly | 90 | 36 | 606 | 2702 | 1.09E-36 | 1.43E-33 |
| GO:0035556 | intracellular signal transduction | 52 | 11 | 644 | 2727 | 1.19E-26 | 7.75E-24 |
| GO:0009190 | cyclic nucleotide biosynthetic process | 41 | 3 | 655 | 2735 | 1.03E-25 | 4.47E-23 |
| GO:0009567 | double fertilization forming a zygote and endosperm | 32 | 2 | 664 | 2736 | 1.36E-20 | 4.44E-18 |
| GO:0009294 | DNA mediated transformation | 31 | 5 | 665 | 2733 | 2.49E-17 | 6.50E-15 |
| GO:0009617 | response to bacterium | 28 | 6 | 668 | 2732 | 9.58E-15 | 1.92E-12 |
| GO:0009611 | response to wounding | 28 | 6 | 668 | 2732 | 9.58E-15 | 1.92E-12 |
| GO:0007165 | signal transduction | 33 | 16 | 663 | 2722 | 8.38E-13 | 1.56E-10 |
| GO:0006182 | cGMP biosynthetic process | 15 | 0 | 681 | 2738 | 3.54E-11 | 5.79E-09 |
| GO:0055085 | transmembrane transport | 35 | 33 | 661 | 2705 | 7.42E-09 | 9.69E-07 |
| GO:0006164 | purine nucleotide biosynthetic process | 9 | 4 | 687 | 2734 | 1.79E-04 | 1.17E-02 |
| GO:0006171 | cAMP biosynthetic process | 8 | 3 | 688 | 2735 | 2.53E-04 | 1.57E-02 |
| GO:0071805 | potassium ion transmembrane transport | 5 | 0 | 691 | 2738 | 3.38E-04 | 2.01E-02 |
| GO:0018298 | protein-chromophore linkage | 6 | 1 | 690 | 2737 | 3.95E-04 | 2.29E-02 |
| GO:0009987 | cellular process | 32 | 58 | 664 | 2680 | 7.38E-04 | 4.10E-02 |
| GO:0008152 | metabolic process | 40 | 82 | 656 | 2656 | 8.36E-04 | 4.46E-02 |
| Core Chlorophyta lineage (2) | |  |  |  |  |  |  |
| GO:0007165 | signal transduction | 37 | 12 | 983 | 2402 | 3.40E-11 | 1.78E-08 |
| GO:0008152 | metabolic process | 61 | 61 | 959 | 2353 | 2.36E-06 | 6.86E-04 |
| GO:0010218 | response to far red light | 14 | 3 | 1006 | 2411 | 1.02E-05 | 2.54E-03 |
| GO:0010114 | response to red light | 15 | 4 | 1005 | 2410 | 1.23E-05 | 2.68E-03 |
| GO:0009637 | response to blue light | 13 | 3 | 1007 | 2411 | 2.86E-05 | 4.98E-03 |
| GO:0055114 | oxidation-reduction process | 61 | 70 | 959 | 2344 | 3.55E-05 | 5.79E-03 |
| GO:0018298 | protein-chromophore linkage | 7 | 0 | 1013 | 2414 | 2.01E-04 | 2.70E-02 |
| Chlorophyta lineage (1) | |  |  |  |  |  |  |
| GO:0035556 | intracellular signal transduction | 61 | 2 | 784 | 2587 | 1.54E-35 | 4.03E-32 |
| GO:0009190 | cyclic nucleotide biosynthetic process | 44 | 0 | 801 | 2589 | 6.79E-28 | 5.91E-25 |
| GO:0006182 | cGMP biosynthetic process | 15 | 0 | 830 | 2589 | 6.68E-10 | 2.91E-07 |
| GO:0006171 | cAMP biosynthetic process | 11 | 0 | 834 | 2589 | 1.91E-07 | 5.53E-05 |

^a^GOD indicates number of retained duplicates with GO. ^b^GON, number of genes that are not retained duplicates with GO. ^c^NGOD, number of retained duplicates without GO. ^d^NGON, number of genes that are not retained duplicates without GO. ^e^p value is calculated using Fisher’s exact test. ^f^FDR value is calculated using R package qvalue. Numbers in parenthesis indicate branches as shown on Figure 2.
