# Supplementary material for: Retained duplicate genes in green alga Chlamydomonas reinhardtii tend to be stress responsive and experience frequent response gains
Source: BMC Genomics. 2015 Mar 4;16(1):149. doi: 10.1186/s12864-015-1335-5 (PMC4364661; doi:10.1186/s12864-015-1335-5)
Supplement: Additional file 8: Table S3. — Gene Ontology categories significantly enriched in conserved genes or genes most closely related to pseudogenes in C. reinhardtii. [file 12864_2015_1335_MOESM8_ESM.doc]

**Supplemental Table 3.** Gene Ontology categories significantly enriched in conserved genes or genes most closely related to pseudogenes in *C. reinhardtii*

| **GO** | **GOG^a^** | **GON^b^** | **NGOG^c^** | **NGON^d^** | ***p*^e^** | **FDR^f^** | **Annotation** |
| --- | --- | --- | --- | --- | --- | --- | --- |
| **Conserved genes** | | |  |  |  |  |  |
| GO:0006412 bp | 27 | 66 | 131 | 4451 | 6.09E-19 | 9.29E-16 | translation |
| GO:0003735 mf | 27 | 67 | 131 | 4450 | 8.31E-19 | 9.29E-16 | structural constituent of ribosome |
| GO:0005840 cc | 26 | 63 | 132 | 4454 | 2.44E-18 | 1.82E-15 | ribosome |
| GO:0005622 cc | 23 | 108 | 135 | 4409 | 3.28E-11 | 1.83E-08 | intracellular |
| GO:0009538 cc | 4 | 1 | 154 | 4516 | 6.12E-06 | 2.74E-03 | photosystem I reaction center |
| GO:0033014 bp | 4 | 2 | 154 | 4515 | 1.79E-05 | 6.66E-03 | tetrapyrrole biosynthetic process |
| **Genes associated with pseudogenes** | | | |  |  |  |  |
| GO:0006950 bp | 34 | 106 | 72 | 5513 | 3.77E-30 | 1.36E-26 | response to stress |
| GO:0005524 mf | 36 | 594 | 70 | 5025 | 1.76E-10 | 3.19E-07 | ATP binding |

^a^GOG indicates number of genes with a particularly GO annotation in the specified group (conserved genes or genes most closely related to pseudogenes). ^b^GON, number of genes with GO not in specified group. ^c^NGOG, number of genes without GO in specified group. ^d^NGON, number of genes without GO not in specified group. ^e^p value is calculated using Fisher’s exact test. ^f^FDR value is calculated using R package qvalue. bp, mf, and cc indicate biological process, molecular function, and cellular components, respectively.
