# Supplementary material for: Retained duplicate genes in green alga Chlamydomonas reinhardtii tend to be stress responsive and experience frequent response gains
Source: BMC Genomics. 2015 Mar 4;16(1):149. doi: 10.1186/s12864-015-1335-5 (PMC4364661; doi:10.1186/s12864-015-1335-5)
Supplement: Additional file 9: Table S2. — C. reinhardtii retained duplicate genes tend to be stress responsive. [file 12864_2015_1335_MOESM9_ESM.doc]

**Supplemental Table 2.** *C. reinhardtii* retained duplicate genes tend to be stress responsive

| **Stress conditions** | **DR^a^** | **DN^b^** | **NDR^c^** | **NDN^d^** | **p^e^** |
| --- | --- | --- | --- | --- | --- |
| N deprivation | 654 | 1156 | 3934 | 10838 | 8.71E-17 |
| S deprivation | 383 | 1324 | 3023 | 11443 | 1.40E-01 |
| Cu deprivation | 48 | 1703 | 196 | 14367 | 3.72E-05 |
| Fe deprivation | 41 | 1722 | 203 | 14439 | 3.42E-03 |
| CO2 deprivation | 106 | 1679 | 567 | 14024 | 8.50E-05 |
| Oxidative stress | 438 | 1438 | 2919 | 12028 | 1.26E-04 |
| all | 1014 | 908 | 7169 | 7858 | 3.37E-05 |
| ^a^DR indicates retained duplicates responsive to stress; ^b^DN, retained duplicates not responsive to stress; ^c^NDR, genes that are not retained duplicates (singletons) responsive to stress; ^d^NDN, genes that are not retained duplicates not responsive to stress; ^e^p, p-value of Fisher’s exact test. | | | | | |
